# Developing a Conceptual Partner Matching Framework for Digital Green Innovation of Agricultural High-End Equipment Manufacturing System Toward Agriculture 5.0: A Novel Niche Field Model Combined With Fuzzy VIKOR

**DOI:** 10.3389/fpsyg.2022.924109

**Published:** 2022-07-08

**Authors:** Shi Yin, Yuexia Wang, Junfeng Xu

**Affiliations:** ^1^College of Economics and Management, Hebei Agricultural University, Baoding, China; ^2^School of Economics and Management, Harbin Engineering University, Harbin, China

**Keywords:** agricultural high-end equipment manufacturing enterprises, digital green innovation, symbiosis theory, field theory, partner selection

## Abstract

Digital green innovation (DGI) is the core factor that affects the digitalization and decarbonization strategy of agricultural high-end equipment manufacturing (AHEM) system. Although AHEM enterprises actively cooperate with academic research institutes to develop agricultural high-end equipment, there are many obstacles in the process of DGI. Moreover, the integration of digital technology and green innovation from the perspective of partner matching for the AHEM system has not been fully introduced in current literature. Hence, this study aimed to (i) establish a suitable framework system for the AHEM system in general, (ii) quantify the selection of DGI by academic research institutions based on niche theory, and (iii) propose an extended niche field model combined with fuzzy VIKOR model. First, a theoretical framework consisting of three core elements of technology superposition, mutual benefit, and mutual trust, and technological complementarity was constructed based on niche intensity and niche overlap degree. DGI ability superposition of technology, mutual trust, and technical complementarity are beneficial for transferring DGI knowledge from academic research institutes to the AHEM industry. Second, triangle fuzzy number and prospect theory combined with the VIKOR method were introduced into the field theory to construct the complementary field model of DGI resources. The niche field model has been successfully applied to practical cases to illustrate how the model can be implemented to solve the problem of DGI partner selection. Third, the results of a case study show that the criteria framework and the niche field model can be applied to real-world partner selection for AHEM enterprises. This study not only puts forward the standard framework of niche fitness evaluation based on niche theory but also establishes the niche domain model of innovation partner selection management based on niche theory. The standard framework and novel niche field model can help enterprises to carry out digital green innovation in the development of high-end agricultural equipment. The study has the following theoretical and practical implications: (i) constructing a criteria framework based on niche theory; (ii) developing a novel niche field model for DGI partner selection of AHEM enterprises; and (iii) assisting AHEM enterprises to perform DGI practice.

## Introduction

Agricultural development is an important guarantee for China to improve its overall economy. China is a big agricultural producer and a big market for agricultural equipment applications. Without agricultural mechanization, there can be no modernization of agriculture and rural areas (Belton et al., 2021). Since the 13th Five-Year Plan, China has made a series of achievements in agricultural mechanization development. The total number of agricultural equipment in China reached 200 million, and the overall mechanization rate of crop cultivation and harvesting exceeded 70%. The production of the three major grains was basically mechanized, China has become the world’s first agricultural equipment producer whose equipment is used by many countries (Qian et al., 2022). Agricultural production mode has realized the historic transformation from mainly relying on human and animal power to relying on mechanical power, aiming to move toward the future development of intelligence (He et al., 2021; Hou et al., 2021). The 2020 China-ASEAN Modern Agricultural Equipment Cooperation and Development Forum was held in Nanning. The forum used “Belt and Road” to promote China-ASEAN agricultural mechanization technology cooperation and exchange. The participants exchanged agricultural equipment production mechanization industry information and scientific research achievements (Aryal et al., 2021). In 2022, the Central No. 1 Document points out that to promote the transformation and upgrading of China’s agricultural equipment industry and strengthen scientific research institutions and equipment manufacturing enterprises to jointly tackle key problems, the development of high-end agricultural machinery and equipment manufacturing is important. Agricultural high-end equipment manufacturing (AHEM) enterprises should improve their ability to independently develop agricultural equipment to support high-end intelligence (Wu et al., 2021; Zein El-Din et al., 2021). The major agricultural modernization project and high-end equipment innovation project were proposed to provide an impetus for green innovation of AHEM enterprises (Wu et al., 2021). The objective is to strengthen the pace of AHEM and provide a strong guarantee to speed up the digital green innovation (DGI) of AHEM enterprises. Agricultural mechanization is an important foundation for transforming the pattern of agricultural development and improving rural productivity and is an important support for implementing the rural revitalization strategy and realizing agricultural modernization (Wei and Cui, 2021). AHEM enterprises should take charge of rural vitalization, with the goal of meeting the needs of agricultural production and the focus on increasing farmers’ incomes. AHEM enterprises should focus on strengthening weak points, promoting coordination, and accelerating the transformation and upgrading of the agricultural equipment industry.

The deep integration of the new generation of information technology and agriculture has given birth to the third green revolution in agriculture (He et al., 2021). The digital revolution of agriculture has brought agriculture into a new era of networking, digitalization, and intellectual development. In the digital revolution of agriculture, two major changes have taken place in world agriculture. One is the emergence of a new mode of agricultural production represented by smart agriculture (Senthil Kumar et al., 2021), which makes agricultural production more “smart” and “intelligent.” The other is the development of an agricultural digital economy activating the value potential of “data elements” and enabling the new development of digital agriculture and rural areas. Smart agriculture is a modern agricultural production mode with information, knowledge, and equipment as the core elements (Senthil Kumar et al., 2021). It is the commanding height of the competition of modern agricultural science and technology and an important direction of the development of modern agriculture. In view of the problems of insufficient interpretation of the connotation of agricultural science, this study further analyzes the selection of key academic research partners for digital green innovation in agriculture (Gibadullin and Sergey, 2021). Agricultural mechanization can improve labor productivity and the utilization rate of land resources and play an “engine” role in agricultural production and management mode. The development of agricultural mechanization should actively cooperate with the implementation of foreign cooperation strategies. The digestion and absorption of foreign advanced agricultural machinery R&D and manufacturing technology can improve China’s effective supply capacity and international competitiveness of modern agricultural equipment (Liu et al., 2020). AHEM enterprises in the process of going out should pay attention to the overall technology, equipment, and other aspects of the group to go out, rather than isolated agricultural machinery to go out. In this process, AHEM enterprises should not only increase their R&D efforts and integrate innovation and opening but also closely combine the innovation of agricultural machinery and equipment with the needs of modern agricultural development (Salembier et al., 2020). To explore the application of information technologies, such as the Internet of Things, big data, mobile Internet, intelligent control, and satellite positioning in agricultural mechanization, AHEM enterprises should actively carry out the automatic navigation of agricultural machinery, precise operation, and unmanned driving and popularize and apply agricultural machinery technology and equipment test demonstration so as to promote agricultural equipment structure optimization and upgrading (Zhang et al., 2022). AHEM enterprises speed up the promotion and application of intelligent monitoring platforms for agricultural machinery operation and promote precise operation (Saiz-Rubio and Rovira-Más, 2020). AHEM enterprises should support the construction of “digital agriculture” demonstration bases and demonstration areas of “unmanned farms.” They help promote the integrated development of intelligent agricultural machinery, “smart agriculture,” and “cloud farms,” and accelerate the promotion of intelligent agricultural machinery services (Xu et al., 2022). Intelligent agricultural machinery and equipment represent the most advanced agricultural productivity. It is the key to improving production conditions, realizing intensive farming, improving production efficiency, changing the development mode, and enhancing the comprehensive production capacity (Li J. et al., 2022). An agricultural mechanization is an important tool for improving labor productivity, land output rate, and resource utilization rate. Intelligent agricultural equipment is the strategic material basis of modern agricultural development and also the focus of technological competition in the international agricultural equipment industry (Tian et al., 2020). Accelerating the development of intelligent agricultural equipment technology is of great significance to improve the supply capacity of agricultural machinery equipment.

In recent years, the development facility of agriculture equipment technology tends to be in a saturated state (Xing et al., 2021). Facilities for agriculture equipment technology level are relatively high (Ma et al., 2021), and these advanced technologies can play more significant stability. When agricultural personnel performs farming tasks, the development of equipment technology is more comprehensive and specific, and the production quality and efficiency are in a high state. Especially in Italy and the Netherlands (Domagała, 2021), no matter in the early stage of cultivation, fertilization, and irrigation in the middle stage, picking and harvesting in the later stage, the application of facility agricultural equipment is very frequent (Domagała, 2021), and the completely artificial agricultural production mode is gradually being replaced. For instance, the Axial fan commonly used in Italy and the curtain motor commonly used in Holland and in some developed countries have been equipped with computer data collection system in the greenhouse with a high degree of automation (Białowąs and Budzyńska, 2022).

At present, China’s AHEM technology has entered a new stage of development. DGI is the future development trend of modern agriculture in China. As the most important part of precision agriculture, the R&D of AHEM is becoming more and more important. Agricultural equipment will integrate engineering technology, advanced manufacturing, and intelligent control of the new generation of information and communication (Domagała, 2021; Białowąs and Budzyńska, 2022). This helps to accelerate the development of high efficiency, intelligent, connection, and green, and become the new demand of modern agricultural development. In recent years, China’s agricultural equipment industry has sustained rapid development and growth. The major domestic agricultural equipment is steadily advancing in the direction of intelligence, high efficiency, and green. The agricultural field is increasingly emphasizing the realization of digitalization, green agriculture, and innovation of agriculture equipment (Garske et al., 2021). Digital green agriculture has become a new trend of agricultural development and is considered the frontier of agricultural science and technology development in the future (Li M. et al., 2022).

Agricultural machinery and equipment are an important foundation for changing the pattern of agricultural development and improving rural productivity (Long et al., 2022), and important support for implementing the rural revitalization strategy. To implement the rural revitalization strategy and promote agricultural and rural modernization, new requirements have been put forward for agricultural mechanization (Long et al., 2022). However, on the whole, the development of AHEM industry is not balanced and sufficient, and some deep-seated contradictions and problems need to be solved (Domagała, 2021; Białowąs and Budzyńska, 2022). First, the effective supply of agricultural machinery equipment is insufficient. There is a lack of stalls and middle- and low-end products overcapacity coexist. The reliability and applicability of machinery and tools need to be further improved. Second, the integration of agricultural machinery and agronomy is not enough (Tang et al., 2021), and the adaptability of variety breeding, cultivation system, planting and rearing methods, post-natal processing, and mechanized production need to be strengthened. Third, the construction of basic conditions lags behind the appropriate mechanization. There are problems, such as agricultural machinery being “difficult to field,” “difficult to work,” and “difficult to store.” Modern agriculture requires relevant farmers to comprehensively apply modern industrial means to promote the construction of productive agriculture (Ali and Dahlhaus, 2022). It is very urgent to realize the maximization of income, integration and efficient development under the control of partial conditions. DGI will become the core feature of future agricultural development, which can comprehensively promote the construction and development of digital green agriculture (Abdulai, 2022). Promoting its modernization is not only important support for improving China’s agricultural production level but also a prerequisite for increasing China’s economic production income. The deep application of facility agriculture can also effectively solve the ecological environment problems and increase the income level of farmers (Lu et al., 2022). The AHEM technology plays a significant role in promoting the construction and development of modern agriculture. The comprehensive integration of agricultural biotechnology, construction engineering technology, agricultural engineering technology, machinery production technology, and other fields of science and technology can greatly promote efficient and high-quality agricultural production (Dayioğlu and Turker, 2021). It is an important path for China’s future agricultural construction and development. But at present, compared with developed countries, China’s research and application of facility agricultural equipment technology are still immature. Therefore, relevant units should actively strengthen the application of relevant programs to provide solid support for China’s agricultural production construction (Zhang and Cai, 2021).

With the rapid development of the agricultural economy and strong support from the state, the innovation of agricultural science and technology has met a golden opportunity and unprecedented challenge. At present, China’s agricultural equipment is still in the stage of extensive development. It is mainly manifested in uneven financial investment in agricultural machinery research, difficulties in advanced technology for R&D, application and promotion of new agricultural machinery products, and heavy and urgent tasks of DGI of agricultural high-end equipment. The development of AHEM enterprises in China is faced with multiple challenges of expanding fields, increasing varieties, and improving functions and levels (Qin et al., 2021). First, large-scale, multi-functional and efficient, innovative, digital, and automated agricultural equipment should be developed. Second, operational technology and equipment for efficient use of resources, such as seed saving, water saving, fertilizer saving, and medicine saving should be developed. Third, digital designing of agricultural equipment, virtual simulation, and integrated lean manufacturing (Khanna, 2021) should be realized. Some AHEM enterprises in China have begun to change their concept and focus on the design, R&D, and production of digital agricultural machinery. Due to a lack of innovative talents, long research periods and high risks, insufficient capital investments, and other factors, it is difficult for AHEM enterprises to realize DGI (Li et al., 2022a,b). AHEM enterprises have to cooperate with partners to carry out DGI activities which have become an inevitable trend. Facing the future, deeply promoting AHE for agriculture, and selecting high-quality partners are particularly important. AHEM enterprises should gather cutting-edge scientific research and integrate superior resources to build an integrated R&D platform. This helps in promoting the formation of agricultural equipment industry technology innovation center.

With the development of green, digital, and intelligent agriculture, it is difficult for AHEM enterprises to rely on traditional partners to carry out DGI activities (Yin et al., 2021a,b). Scholars have studied this from different perspectives and put forward some solutions. In terms of research topics, partner selection was studied from the perspective of technology standardization, collaborative innovation, and cooperative network, respectively (Wang et al., 2015; Han et al., 2019; Guertler and Sick, 2021). Choi (2020) studied the influence of knowledge protection on alliance partnership selection from the perspective of knowledge management. Li et al. (2020) and Li and Yin (2020) respectively studied the problem of ecological partner selection. Jäger and Piscicelli (2021) collaboratively studied the partner selection process for circular food packaging. Ávilaey et al. took virtual enterprises as the research subject and used different methods to study the partner selection of virtual enterprises. In terms of research methods, the virtual enterprises mainly focus on the multi-attribute decision making method (Mousavi and Gitinavard, 2019; Zeng et al., 2019), network correlation analysis method (Chen et al., 2021), SEM method (Hair et al., 2019), VIKOR group decision making method (Wang et al., 2019), mathematical analysis method (Bolandnazar et al., 2020), intuitionistic fuzzy theory (Yang et al., 2021), method set (Venkatesh et al., 2019), and stage method (Ávila et al., 2021).

The above literature has carried out a comprehensive and in-depth study on ecological partner selection from the aspects of the research subject, research perspective, and research methods. This provides a valuable reference for the selection of academic research partners for AHEM enterprises. However, in terms of research subjects and perspectives, there are few studies on the selection of academic research partners of AHEM enterprises. Due to financing constraints, there are only a few studies on the selection of academic research partners for the digital innovation of enterprises. Most of the research methods have a single evaluation method, which may lead to problems in the evaluation results. Even if multiple methods are integrated, most of the selected partners are only assessed from the evaluation value. The partners to be selected lack investigation from the complementarity of resources and the rationality and matching of partner selection. The continuity of the interaction between partner selection subject and partner is ignored. Therefore, it is necessary to conduct further research on the selection of academic research partners of AHEM enterprises to improve the accuracy of partner selection. This paper provides a new way of thinking about how to improve the scientificity and rationality of cooperative partner selection for AHEM enterprises.

This study focuses on the research on the partner selection of digital green innovation mutualism for AHEM enterprises. A niche fitness evaluation standard framework based on niche theory was proposed and a niche field model for innovation partner selection management based on niche theory was established. The standard framework and novel niche field model can help enterprises carry out DGI in the development of high-end agricultural equipment. The study has the following theoretical and practical implications: (i) constructing a criteria framework based on niche theory; (ii) developing a novel niche field model for DGI partner selection of AHEM enterprises; (iii) assisting AHEM enterprises to perform DGI practice. This helps optimize the selection of academic research partners and strengthen the cooperation between AHEM enterprises and academic research partners. This study can provide direction and strategy for improving the transformation effect of DGI in AHEM enterprises.

The rest of the study is organized as follows. Section ^***^”Literature Review and Theoretical Framework” is a literature review and theoretical framework. The Niche field model based on niche theory and field theory is shown in Section “Methodology.” Section “Empirical Study” is an empirical study. Conclusions and future prospects are presented in Section “Conclusion and Implications.”

## Literature Review and Theoretical Framework

### Literature Review

#### Digital Green Innovative Management

Digital green innovative management involves digital innovation, green innovation, and the integration of DGI. Digital innovation can promote high-quality development of agricultural high-end equipment. Räisänen and Tuovinen (2020) thought that rural digital infrastructure construction is very important to develop the rural digital economy and promote the pilot development model of smart agriculture. Nam et al. (2021) believed that digital technology should be actively explored to lead the innovative development of rural agriculture and give way to the guiding role of digital technology to drive the overall innovation of rural agriculture. Haggag (2021) proposed the rational application of digital technology in rural agricultural construction and development and constructed the development model of digital agriculture. Tang et al. (2020) found that digital financial development has a “structural” driving effect on enterprise technological innovation. Liu et al. (2021) explored the internal mechanism of digital technology enabling high-quality agricultural development in China by embedding digital technology into the agricultural factor allocation system and industrial system. Maria et al. (2021) explored the construction of digital agriculture operation management theory and methods to promote China’s modern agriculture from mechanization and electrification to digital and intelligent leaps. Cao et al. (2021) enriched relevant studies on the impact of digital finance on enterprise innovation and provided a theoretical basis and policy reference for how the financial market can better serve the real economy. Tang et al. (2022) put forward the concept of enterprise strategic alliance ecosphere in the era of “Internet +” and discussed the innovation paradigm of enterprise strategic alliance ecosphere.

Green innovation has promoted the green development of AHEM enterprises. Cao et al. (2012) developed green agriculture through technological progress and promoted the green transformation of agricultural development policies, production organizations, technical services, and management systems. Wang and Liu (2015) studied how manufacturing enterprises design their supply chain structure from the perspective of environmental strategy and effectively identified the partnership between supply chain manufacturers. Xie et al. (2015) analyzed the mechanism of green development of enterprises and pointed out the path of green development of enterprises in the context of digitization. Reid and Miedzinski proposed that enterprise green innovation has the function of value symbiosis and encouraged enterprises to take the initiative in green innovation. Guo (2017) explored the topic of green financial services agriculture, rural areas, and farmers and built a green financial service system to promote the development of green agriculture. Lin (2019) believed that rural revitalization adheres to the orientation of sustainable development. Yue et al. (2020) found that the Internet has a positive impact on green innovation performance and promotes the green innovation development of manufacturing enterprises. Yin et al. (2020a) found that digital finance improves regional green innovation efficiency and affects the green innovation efficiency of neighboring regions, and the rural society must carry out green financial innovation. Fang et al. found that the greener the patent applications and licenses a GeM-listed company can obtain, the higher the excess return rate of its stock. Zhang et al. (2021) studied how to effectively drive agricultural enterprises to carry out green entrepreneurship, which is conducive to deepening the research on green entrepreneurship of agricultural enterprises. The integration of DGI has opened up a new way for the innovative development of agricultural high-end equipment. Zhai and Lewis (2021) found that the incentive effect of digital financial development on green innovation of enterprises is more significant in the central and western regions with lower development levels. Li et al. explored the impact of different digital innovations on green innovation performance, which has important theoretical and practical significance. Yu and Yang (2021) studied the influence mechanism between digital finance and enterprise green innovation in terms of regional heterogeneity. Lei et al. (2021) found that infrastructure construction, market competition intensity, and enterprise innovation input have a significant positive impact on the ecological benefits of DGI in the manufacturing industry. Han and Gan (2022) proposed that investors’ attention can promote green innovation performance of enterprises. Wei et al. (2022) found that the digital economy effectively improves the output of urban green innovation.

At present, most literature research subjects are mainly concentrated on other enterprises, and there are few studies on AHEM enterprises. Research on the partner selection of DGI for AHEM enterprises is less based due to financing constraints.

#### Partner Selection Guidelines

Many scholars have been engaged in the research of partner selection criteria, which mainly focus on green innovation, digital innovation, ecological innovation, industry-university-research innovation, and other partner selection. In terms of green innovation partner selection criteria, Kang et al. (2015) used the multi-objective optimization model to quantitatively analyze and study the partner selection problem of cloud service providers. Wang and Liu (2016) constructed a supplier evaluation and selection index system by using carbon dioxide emissions as a key indicator of supply chain environmental performance. Li et al. (2020) selected the degree of cooperation aggregation, data and information security protection ability, cooperation transformation ability, cooperation embedding ability, and aggregation resource level as the partner evaluation indicator system. Zhou et al. constructed an evaluation index system for collaborative innovation partners in cloud manufacturing based on four first-level indicators, namely compatibility, innovation capability, technical scheme, and innovation effect. Mutamimah et al. (2021) constructed a 3 C principle of compatibility, capability, and commitment. Li M. et al. (2022) used big data of enterprise patent cooperation to measure the behavior of innovation partner selection among enterprises.

In terms of selection criteria for eco-innovation partners, Zhang et al. (2013) took environmental protection as an important indicator to evaluate partners and added carbon and lead content as environmental indicators into the partner evaluation system. Zhang et al. (2014) proposed a set of science and technology collaborative partner evaluation index system for the global supply chain system environment. Zhai and Zhao constructed evaluation index system criteria for partner selection under the ecosystem platform and established a model for partner selection of the ecosystem based on the Markov chain. Li and Yin (2020) put forward the evaluation model and selection model of ICT enterprise ecological partner selection. Hua and Anna (2021) studied the selection of ideal partners from the perspective of the resource investment rate of each subject, the degree of resource complementarity, and the comparison of comprehensive strength. Wang et al. (2022) proposed three types of alliance: win-win alliance, unilateral alliance, and unintentional alliance partner.

In terms of the selection criteria of industry-university-research partners, Zhu et al. (2014) evaluated the selection of cooperative innovation partners based on subjective criteria, such as knowledge possession, management experience, technical ability, and resource complementarity. Yu and Hu (2015) and Yang and Li (2016) established an index system from the resource ability, management ability, technical skills, property rights, reputation, and the degree of compatibility to study partner selection, respectively. Dao et al. (2014) and Huang et al. (2017) studied partner selection from the aspects of cost, time, quality, risk, and reputation. Chen et al. (2018) considered indicators, such as security and synergy. Ma et al. (2018) compared the matching of industry-university-research partners, target synergy, cultural compatibility, innovation resources, and complementarity of capabilities as the selection criteria to investigate the collaborative capabilities of partners. Zheng et al. used expert evaluation and system analysis methods to conduct a comprehensive evaluation on the selection of partners. Chen et al. (2021) summarized that quality and capability are the main indicators for the evaluation of industry-university-research collaborative innovation partners. Su et al. (2022) found that core members will preferentially select collaborative members with knowledge stock advantages to form a knowledge supply chain.

The research on influencing factors of partner selection is the construction of an evaluation index system for partner selection. Moreover, the research focuses on two aspects of task-oriented factors and relationship-oriented factors. The results are highly consistent, indicating that the importance of some factors in partner selection has reached a consensus.

#### Partner Selection Method

In terms of research methods, the partner selection method mainly focuses on the multi-attribute decision making method (Mousavi and Gitinavard, 2019; Zeng et al., 2019), network correlation analysis method (Chen et al., 2021), SEM method (Hair et al., 2019), VIKOR group decision making method (Wang et al., 2019), mathematical analysis method (Bolandnazar et al., 2020), intuitionistic fuzzy theory (Yang et al., 2021), method set (Venkatesh et al., 2019), and stage method (Ávila et al., 2021). For example, Mousavi and Gitinavard (2019) extended a multi-attribute group decision approach for the selection of outsourcing service activities for information technology under risk. Wang et al. (2019) investigated a fuzzy AHP-VIKOR based prioritization from a life cycle perspective to select sustainable energy conversion technologies for agricultural residues. Venkatesh et al. (2019) adopted a fuzzy AHP-TOPSIS approach to investigate supply partner selection. Hair et al. (2019) established a structural equation modeling-based discrete choice modeling to study the retailer choice problem. Bolandnazar et al. (2020) used artificial intelligence and mathematical models to study energy consumption forecasting in agriculture. Li et al. (2020) developed an ecological partner selection field model of strategic alliance based on dual combination weighting. Yang et al. (2021) used an intuitionistic fuzzy weighted approach to integrate individual criteria decision matrix of partners in different periods to achieve a continuous evaluation of partners. Yang and Wang (2022) constructed a partner selection evaluation index system.

The above analysis results lay a foundation for further analysis in this study. However, it is found that the existing research has still deficiencies in the following three aspects. First, few scholars take the factors of mutual trust and technology accumulation into consideration in the analysis of influencing factors. Different characteristics of different innovation types will lead to different factors to be considered in partner selection. The technological level of the partner has also an important influence on the selection of a partner. Second, existing studies only simply analyze the factors influencing corporate partner selection, without an in-depth analysis of the mechanism among the factors, which has a limited guiding effect on practice. Third, simple correlation analysis or speculative research is generally adopted, with few empirical studies. To remedy the above research defects, this study analyzes the selection of academic research partners for DGI of AHEM enterprises. It provides decision-making guidance for optimizing the management of the selection of academic research partners. In addition, this study provides a direction strategy for the selection of cooperative partners of AHEM enterprises.

### Theoretical Framework

**FIGURE 1:**
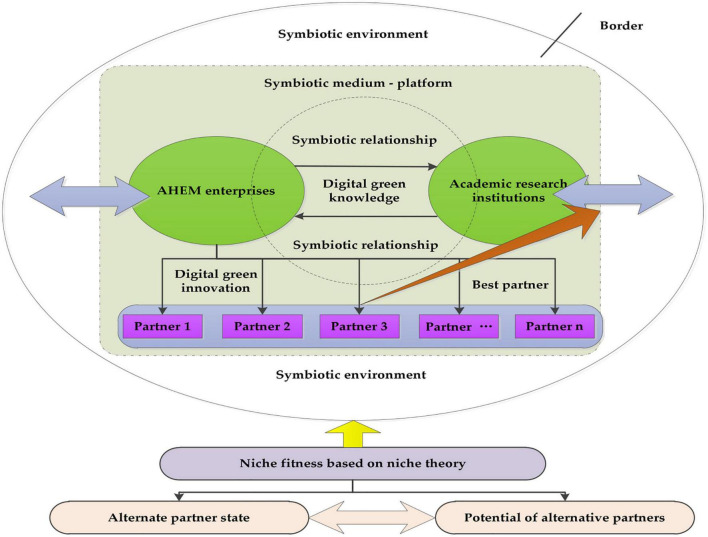
A theoretical model of DGI partner selection.

Innovation usually occurs in the process of technological change. High-end equipment innovation plays an important role in improving the competitiveness of AHEM enterprises. It is regarded as one of the key factors affecting the green competitive advantage and strategic selection of AHEM enterprises. In AHEM R&D process, enterprises are faced with huge digital technology and green technology problems. In recent years, more and more agricultural high-end equipment is developed by enterprises, universities, and scientific research institutes on the basis of knowledge and technology sharing and cooperation in obtaining complementary resources (Tuna and Karantininis, 2021). DGI problems related to agricultural high-end equipment R&D can be solved by choosing ideal partners. Figure 1 shows the theoretical model of partner selection for DGI cooperation of AHEM enterprises.

In Figure 1, the two main bodies of high-end equipment innovation are AHEM enterprises and academic research institutions. DGI cooperation and technology sharing between AHEM enterprises and academic research institutions can promote DGI. Cooperation between AHEM enterprises and academic research institutes can not only make enterprises share R&D costs and innovation risks with academic research institutes but also realize knowledge and technology sharing and obtain complementary resources (Yin et al., 2020b,c). The cooperation not only combines heterogeneous partners but also heterogeneous knowledge more importantly. In the process of exchanging resources, the mutually beneficial and symbiotic relationship between AHEM enterprises and academic research institutions is gradually formed. More and more complementary resources of DGI are shared between AHEM enterprises and other academic research institutions. Whether AHEM enterprises can choose appropriate mutually beneficial partners is directly related to the development of DGI. In the selection system, it is particularly important for AHEM enterprises to select one or several academic research institutions as their partners for DGI of agricultural high-end equipment. This study will solve this issue.

**FIGURE 2:**
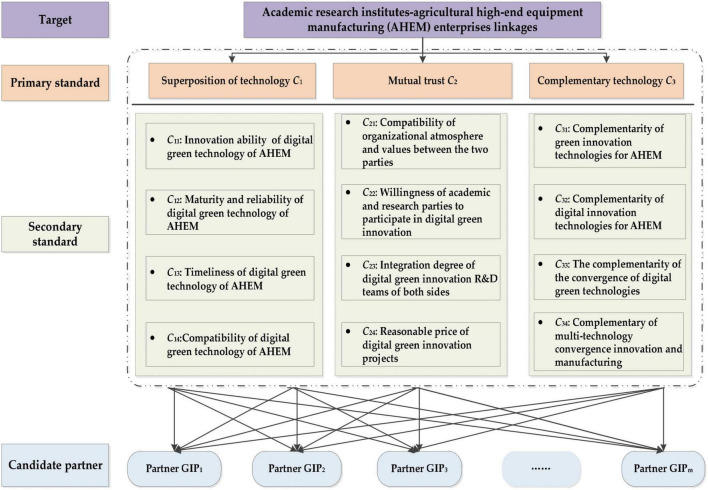
The evaluation system of academic research institutions selection of DGI based on niche theory.

In this study, a standard framework for the selection of academic research partners for the DGI cooperation of AHEM enterprises is constructed. Every academic research institution has the niche theory of state attribute and potential attribute. The state attribute refers to the unit of an organism, its past growth, development, and interaction with the environment, while potential refers to the actual influence or dominance of the unit of an organism on the environment and reflects its development trend. From the perspective of niche theory, niche intensity and niche overlap are the core contents of niche state and potential. In the collaborative DGI system, niche intensity is the state of the academic research institutes and the result of the interaction between growth, development, and innovation environment in the past. The degree of niche overlap is the actual influence or dominant position of academic research institutions on AHEM enterprises. Figure 2 shows the evaluation system of academic research partner selection of DGI based on Figure 1.

The characteristics and capabilities of academic research institutions are the core content of niche intensity. The characteristics are relatively stable. It is based on the mutual trust of the academic research institutions, such as the compatibility of the atmosphere and values of the cooperative organizations of both sides, the willingness of the academic research institutions to participate in DGI, the integration of the DGI R&D teams of both sides, and the reasonable price of DGI projects. The capability reflects the dynamic characteristics of academic research institutions, such as innovation ability, maturity, and reliability of digital green technology of AHEM, timeliness of digital green technology of AHEM, compatibility of digital green technology of AHEM with enterprise technology, etc. The complementarity of green innovation technology of AHEM, the complementarity of digital innovation technology of AHEM, the complementarity of digital technology and green technology fusion, and the complementarity of multi-technology fusion innovation and manufacturing are the core contents of the ecological niche overlap degree of AHEM enterprises and academic research institutions. Based on the above analysis, mutual trust and technology overlay can be used to reflect the intensity of the niche. The level of complementarity can be used to reflect the degree of niche overlap in the DGI system of academic research institutes-AHEM enterprises linkages.

## Methodology

### Preliminary

#### Triangular Fuzzy Number

With the development of fuzzy theory, the description and expression of fuzziness are more and more demanding. Among many fuzzy numbers, triangular fuzzy numbers (TFNs) are widely used in control, decision making, evaluation, and other fields. In this study, TFNs were adopted to obtain the cooperative intention of the academic research institutions based on the evaluation system. Based on the above analysis, we made the following definition.

**Definition 1:** Let *_Ã=(a^L^,a^M^,a^R^)_* a typical TFN, and its form is as follows:


(1) $$\mu_{\tilde{A}}\,\left(x\right)=\left\{ \begin{array}{c} \frac{x-a^{L}}{a^{M}-a^{L}},a^{L}\leq x\leq a^{M}\\ 0,o\,t\,h\,e\,r\,w\,i\,s\,e\\ \frac{a^{R}-x}{a^{R}-a^{M}},a^{M}\leq x\leq a^{R} \end{array}\right.$$

In this study, fuzzy language variables as shown in Table 1 are used to reflect the evaluation of academic research institutes-AHEM enterprise linkages toward agriculture 5.0.

**TABLE 1:** Fuzzy linguistic variable.

Linguistic variables	Abbreviations	TFNs
Very poor	VP	(0.0, 0.0, 0.1)
Poor	P	(0.0, 0.1, 0.3)
Medium poor	MP	(0.1, 0.3, 0.5)
Medium	M	(0.3, 0.5, 0.7)
Medium good	MG	(0.5, 0.7, 0.9)
Good	G	(0.7, 0.9, 1.0)
Very good	VG	(0.9, 1.0, 1.0)

**Definition 2:** A TFN *_Ã=(a^L^,a^M^,a^R^)_* is transferred into a crisp real number by:


(2) $$P\,\left(\tilde{A}\right)=\frac{1}{6}\,\left(a^{L}+a^{M}+a^{R}\right)$$

**Definition 3:** Supposing two TFNs *_Ã=(a^L^,a^M^,a^R^)_* and *_$\tilde{B}$=(b^L^,b^M^,b^R^)_* the distance of the two TFNs is calculated by:


(3) $$d\,\left(\tilde{A},\tilde{B}\right)=\frac{\sqrt{3}}{3}\,\sqrt{{\left(a^{L}-b^{L}\right)}^{2}+{\left(a^{M}-b^{M}\right)}^{2}+{\left(a^{R}-b^{R}\right)}^{2}}$$

#### Prospect Theory

Prospect theory is an effective tool to reflect intuitively perceived utility. In this study, fuzzy prospect theory was introduced to help AHEM enterprises avoid risks blindly or like risks. If the number of academic research institute partners is odd, the median is taken as the reference point. If the number of academic research institute partners is even, the mean of the two fuzzy numbers in the middle is taken as the reference point. Let the reference point of criterion value under the state _*S_1_*_ in criterion _*c_1_*_ be _*Y_jh_*_, and the prospect value function can be determined as follows:


(4) $$v\,\left(y_{i\,j\,h}\right)=\left\{ \begin{array}{c} {\left[d\,\left(y_{i\,j\,h},Y_{j\,h}\right)\right]}^{\alpha }\;y_{i\,j\,h}\geq Y_{j\,h}\;\\ -\lambda \,{\left[d\,\left(y_{i\,j\,h},Y_{j\,h}\right)\right]}^{\beta }\;y_{i\,j\,h}<Y_{j\,h} \end{array}\right.$$

Where _α,β_ is the coefficient of risk attitude, _α,β∈[0,1]_. When _α=β=1_, decision-makers are regarded as risk neutral. Here, we define _α=β=0.88_. _λ_ is the loss avoidance coefficient and define _λ=2.25_. Decision weights are closely related to objective probability. Therefore, the ratio of the weight with the probability of occurrence _*p*_ to the deterministic weight is taken as the decision weight of gain and loss, which can be expressed as follows:


(5) $$\pi \,\left(p_{j}\right)\,\left\{ \begin{array}{c} \pi^{+}\,\left(p_{j}\right)=p_{j}^{\gamma }\,/\,{\left[p_{j}^{\gamma }+{\left(1-p_{j}\right)}^{\gamma }\right]}^{1/\gamma }\\ \pi^{-}\,\left(p_{j}\right)=p_{j}^{\delta }\,/\,{\left[p_{j}^{\delta }+{\left(1-p_{j}\right)}^{\delta }\right]}^{1/\delta } \end{array}\right.$$

Where γand δ represent the risk attitude coefficient of gain and loss respectively, and we define γ=0.61 andδ = 0.69. Then the comprehensive prospect value matrix can be expressed as follows:$V={\left[v\,\left(a_{i\,j}\right)\right]}_{m\times n}={\left[\sum_{h=1,v\,\left(y_{i\,j\,h}\right)\geq 0}^{l}v\,\left(y_{i\,j\,h}\right)\,\pi^{+}\,\left(p_{j}\right)+\sum_{h=1,v\,\left(y_{i\,j\,h}\right)<0}^{l}v\,\left(y_{i\,j\,h}\right)\,\pi^{-}\,\left(p_{j}\right)\right]}_{m\times n}$.

#### VIKOR Method

The measurement criterion of the VIKOR method is developed from the functional form of the compromise programming method. The uncertainty of the partner selection process and the fuzziness of decision-making process can be effectively solved by the VIKOR method.

**Step 1:** Set *f*^+^ as the positive ideal point of the attribute, *f*^−^ as the negative ideal point of the attribute, then


(6) $$\begin{array}{c} f_{j}^{+}=\left\{\max_{i}v\,\left(a_{i\,1}\right),\max_{i}v\,\left(a_{i\,2}\right),\cdots ,\max_{i}v\,\left(a_{i\,m}\right)\right\}\\ f_{j}^{-}=\left\{\min_{i}v\,\left(a_{i\,1}\right),\min_{i}v\,\left(a_{i\,2}\right),\cdots ,\min_{i}v\,\left(a_{i\,m}\right)\right\} \end{array}$$

**Step 2:** The *S*_*i*_ and *R*_*i*_ could be computed by:


(7) $$S_{i}=\sum_{j=1}^{l}w_{j}\,\left(\frac{f_{j}^{+}-v\,\left(a_{i\,j}\right)}{f_{j}^{+}-f_{j}^{-}}\right),R_{i}=\max_{j}\left\{w_{j}\,\left(\frac{f_{j}^{+}-v\,\left(a_{i\,j}\right)}{f_{j}^{+}-f_{j}^{-}}\right)\right\}$$

**Step 3:** The values *Q*_*i*_ could be calculated by:


(8) $$Q_{i}=\theta \times \frac{S_{i}-\min_{i}S_{i}}{\max_{i}S_{i}-\min_{i}S_{i}}+\left(1-\theta \right)\,\frac{R_{i}-\min_{i}R_{i}}{\max_{i}R_{i}-\min_{i}R_{i}}$$

Where θ is a weight parameter.

#### Combined Weight Method

(1) Entropy weight method

The entropy weight method is an objective way to determine weights according to the basic principle of information theory. The specific steps are as follows.

**Step 1:** Standardize the original decision matrix can be expressed as follows:


(9) $$r_{i\,j}=\left\{ \begin{array}{c} x_{i\,j}^{L}/\max_{i}x_{i\,j}^{R},x_{i\,j}^{M}/\max_{i}x_{i\,j}^{M},x_{i\,j}^{R}/\max_{i}x_{i\,j}^{L}\,\wedge \,1\\ \min_{i}x_{i\,j}^{L}/x_{i\,j}^{R},\min_{i}x_{i\,j}^{M}/x_{i\,j\,h}^{M},\min_{i}x_{i\,j}^{R}/x_{i\,j}^{L}\,\wedge \,1 \end{array}\right.$$

**Step 2:** The entropy of each criterion is calculated by:


(10) $$e_{j}=-\frac{1}{\ln m}\,\sum_{i=1}^{m}p_{i\,j}\,\ln p_{i\,j},p_{i\,j}=\frac{r_{i\,j}}{\sum_{i=1}^{m}r_{i\,j}},0\,\ln0=0$$

**Step 3:** The normalized criteria weight is computed by:


(11) $$\alpha_{j}=\left(1-e_{j}\right)\,/\,\sum_{j=1}^{n}\left(1-e_{j}\right)$$

(2) Analytic hierarchy process method

The analytic hierarchy process (AHP)_ is a subjective weighting method that has strong applicability and operability. In addition, all elements under the same criterion layer are compared in pairs by a 1–9 scale method combined with an expert consultation method. Finally, the weights of each factor layer and criterion layer β_*j*_ are calculated (Yin et al., 2022).

(3) Combination weight

The subjective weight β_*j*_ is obtained by AHP, and the objective weight α_*j*_ is obtained by the entropy weight method. To make the combination weight as close as possible to the subjective and objective weights, the combination weight can be expressed as follows:


(12) $$\left\{ \begin{array}{c} \min F=\sum_{j=1}^{n}w_{j}\,\left[\ln w_{j}-\ln\alpha_{j}\right]+\sum_{j=1}^{n}w_{j}\,\left[\ln w_{j}-\ln\beta_{j}\right]\\ s.t.\sum_{j=1}^{n}w_{j}=1,w_{j}>0 \end{array}\right.$$

The Lagrange multiplier method is used to get the combined weight *w*_*j*_:


(13) $$w_{j}=\frac{{\left(\alpha_{j}\,\beta_{j}\right)}^{1\,/\,2}}{\sum_{j=1}^{n}{\left(\alpha_{j}\,\beta_{j}\right)}^{1\,/\,2}}$$

### Niche Field Model Based on Niche Theory and Field Theory

#### Concept and Definition of Niche Field Model

Niche is an extremely important concept in ecology and one of the most important basic theories of ecology. In ecology, different niches have different levels. When agricultural high-end equipment market opportunities arise, AHEM enterprises first respond to this opportunity. AHEM enterprises first use their DGI resources to occupy resource space. When their resources are insufficient to fully occupy the resource space, they have to seek a partner to cooperate with DGI. From the perspective of resource complementarity, a complementary matrix is constructed based on the level of DGI resources of AHEM enterprises and academic research institutes in the niche field model. A niche field is introduced to reflect resource space consisting of all DGI resources, and the field source _*O*_ represents AHEM enterprises that first respond to a certain market opportunity. The niche-fitness of academic research institutes can be transformed into the radius of a niche field based on niche-fitness, namely _*R*_, which reflects the niche-fitness distance between AHEM enterprises and academic research institutes. In the niche field, the resultant force of academic research institutes is determined by the interaction between attraction and resistance of academic research institutes, namely _*F_a_*_ and _*F_r_*_. As time goes by, the value of _*R*_, _*F_a_*_, and _*F_r_*_ change dynamically, and then DGI partners should be dynamically selected or eliminated. In this study, the novel niche field model considering resource complementarity is developed to select the best academic research institute for DGI of AHEM enterprises.

#### Niche-Fitness Mass

Niche-fitness mass of AHEM enterprises and academic research institutes is determined by resource vector and resource utilization ratio. In *N*-dimensional resource space, the resource vector of AHEM enterprises is expressed as *P* = (*p*_1_,*p*_2_,⋯,*p*_*n*_), where *n* represents the dimension of the resource space, *p*_*i*_ = 1 represents that this innovation resource meets the requirement for DGI, while *p*_*i*_ = 0 represents that this innovation resource cannot meet the requirement of DGI. The resource utilization ratio is expressed as *Y* = (*y*_1_,*y*_2_,⋯,*y*_*n*_). *y*_*i*_ ∈ [0,1] and represents the availability of *p*_*i*_. Due to market demand and other factors, the DGI resources will change dynamically at different times. Hence, the time vector *T* is introduced into the niche field model, where *T* = (*t*_1_,*t*_2_,⋯,*t*_*n*_). The niche-fitness mass of AHEM enterprises is calculated by:


(14) $$M_{T}=P_{T}\times Y_{T}=\sum_{i=1}^{n}p_{i}\,y_{1\,i}$$

The resource vector of AHEM enterprises is expressed as *Q* = (*q*_1_,*q*_2_,⋯,*q*_*n*_). Resource space saturation vector and resource demand vector, namely $P_{m}=\overset{n}{\overbrace{\left(1,1,\cdots ,1\right)}}$ and $\bar{P}$. The niche fitness mass of academic research institutes is calculated by:


(15) $$m_{T}=\left(\left(P_{m}⊕P\right)\,⋂Q\right)=\sum_{i=1}^{n}\left[\left(1⊕p_{i}\right)\,\wedge \,q_{i}\right]\,y_{2\,i}$$

#### Field Strength and Attraction

The field strength of the niche field reflects the influence intensity of the AHEM enterprises on the academic research institutes. The field strength *E* is expressed as:


(16) $$E_{T}=\delta \,K\,\frac{M_{T}}{R_{T}^{2}}$$

Where δ represents the parameter that affects the niche field environment, δ ∈ [0,1]. *K* represents the niche effect produced by AHEM enterprises and academic research institutes. Let *Z*_*T*_ be the mass increment; it is calculated by:


(17) $$K_{T}=\frac{Z_{T}}{M_{T}+m_{T}}$$

Attraction reflects the recognition or attraction of AHEM enterprises to academic research institutes in a niche field, and the attraction is calculated by


(18) $$F_{r\,T}=E_{T}\times m_{T}=\frac{Z_{T}\,M_{T}\,m_{T}}{R_{T}^{2}\,\left(M_{T}+m_{T}\right)}$$

#### Radius of a Niche Field

The radius of the niche field reflects the niche-fitness distance between AHEM enterprises and academic research institutes. Let *C*_*f*_ be the value of the character, ability, and compatibility of AHEM enterprises, and academic research institutes. Let *C* be the quality and capability of the research institution. The radius of the niche field is calculated by


(19) $$R_{T}=1+C_{f}-C,C\in \left[0,1\right]$$

The radius of the niche field is calculated by *R*_*T*_=2–*C*. The radius of the niche field in this study is divided into four levels shown. The four levels of the radius include high niche-fitness (0, *R*_*1*_], medium niche-fitness (*R*_*1*_, *R*_*2*_], low niche-fitness (*R*_*2*_, *R*_*3*_], and zero niche-fitness (*R*_*3*_, ∞).

Resistance of academic research institutes reflects the opportunity cost and risk cost of academic research institutes joining the AHEM system, where and represent the opportunity cost and the risk cost, respectively. It is calculated by:


(20) $$F_{r}=D_{1}+D_{2}$$

#### Dynamic Analysis of Niche Field

To improve DGI ability, AHEM enterprises should eliminate one or more academic research institutes that cannot meet the standards of character, ability, compatibility, and resource complementarity, and select one or more partners that meet their requirements. In this process, the niche-fitness mass, field strength, and radius have also been changed.

(1) Let *Q*_*t*_ denote the niche-fitness mass of academic research institutes at a time *t*, and the new niche-fitness mass is calculated by


(21) $$M_{t+1}=M_{t}+m_{t}-Q_{t}$$

(2) The field strength of niche field has also been changed, and the new field strength is calculated by


(22) $$E_{t+1}=K_{t+1}\,\frac{M_{t}+m_{t}-Q_{t}}{R_{t+1}^{2}}$$

At the same time, the circle density of the niche field has also been changed.

**FIGURE 3:**
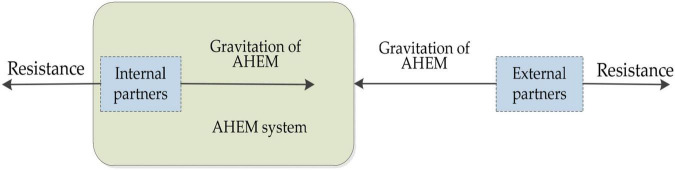
The internal and external forces of DGI partners.

(3) The resistance of willingness to cooperate has changed or even multiplied. At this time, the resistance of willingness is expressed as *F*_(*t* + 1)*W*_ = η*M*_*t* + 1_. The external partners of the AHEM system are also affected by the interaction between attraction*F*_*TG*_ and resistance *F*_*TW*_ and are shown in Figure 3.

## Empirical Study

### Empirical Background

Chenguang Biotech Group Co., Ltd founded in 2000 is a collection of intensive processing of agricultural products and natural plant extraction as one of the export earning enterprises located in Quzhou County, Hebei Province. With advanced equipment and technology and a scientific and efficient technology innovation system, the company owns more than 20 national patented technologies and has built the first provincial natural pigment engineering technology research center in the industry. The technology center of the company has been identified as the national enterprise technology center. At present, one of the important issues is how to develop AHEM and enhance capacity for DGI. The AHEM enterprise needs to select an appropriate DGI partner from these academic research institutions for carrying out the DGI activities of AHEM. In recent years, there are seven academic research institutes carrying out DGI innovation activities, and one or more partners should be selected from these academic research institutions for the AHEM enterprise. In addition, all these academic research institutes are willing to carry out the DGI activities of AHEM.

Although the AHEM enterprise has some experience in partner selection, it is still a difficult problem for the AHEM enterprise to select the best DGI partner. However, the complementary resources used to innovate the AHEM are gradually valued by the AHEM enterprise experts and have become an important factor influencing the success rate of the AHEM enterprise. Moreover, it is difficult to accurately describe the real situation of academic research institution partners by defining real numbers. In fact, exact numbers cannot be used to accurately reflect the real situation of academic research institutions. In the study, the rationality and matching of resource complementarity and partner selection are developed to select the best academic research institute based on the niche field theory.

### Empirical Elements

#### Development of Evaluation Criteria

In order to evaluate the niche fitness of candidate DGI partners, niche fitness is introduced. The theory has a solid theoretical foundation and can meet the requirements of digital innovation and green innovation. The criterion of niche fitness evaluation usually comes from niche strength and niche overlap degree. In the AHEM system, niche strength is the state of academic research institutes, and is the result of past growth, development, and interaction with the innovation environment. The degree of niche overlap is the actual influence or dominance of academic research institutes on AHEM enterprises and reflects their collaborative development trend. Figure 2 illustrates the criteria framework.

#### Data and Scenarios

In the study, ten members in two expert panels are randomly selected from the four related fields: digital innovation, green innovation, business cooperation, and AHEM. These experts are divided into two expert panels. The first expert panel includes five members focusing on DGI, while the second expert panel includes five members focusing on AHEM. The language variables used to evaluate the comprehensive level of academic research institutions are shown in Table 1. The score matrix of academic research institutions provided by DGI experts is shown in Table 2, and the matrix provided by AHEM experts is presented in Table 3.

**TABLE 2:** The evaluation matrix of academic institutions provided by DGI experts.

	C11	C12	C13	C14	C21	C22	C23	C24	C31	C32	C33	C34
GIP1	MP	G	M	P	M	MG	M	M	VG	G	P	M
GIP2	P	P	G	M	VG	M	P	MG	M	VP	VP	MP
GIP3	M	VP	MG	G	MP	P	MG	P	M	MP	MG	VP
GIP4	MG	M	M	VG	MG	G	VP	VP	VP	VG	VG	M
GIP5	VG	G	P	MP	G	MP	G	M	MP	G	M	G
GIP6	G	VG	VP	G	M	G	M	M	MG	M	MP	MG
GIP7	MP	P	MP	MG	MP	VG	G	VG	M	MP	G	VP

**TABLE 3:** The evaluation matrix of academic institutions provided by AHEE experts.

	C11	C12	C13	C14	C21	C22	C23	C24	C31	C32	C33	C34
GIP1	VP	VG	G	MP	P	G	G	G	MG	MG	M	G
GIP2	MP	MP	M	G	G	MP	M	MG	M	M	VP	M
GIP3	G	P	VG	MG	M	M	M	P	MP	MP	MG	MP
GIP4	M	G	P	M	P	MG	P	VP	M	G	VG	G
GIP5	MG	M	MP	P	VG	M	MP	P	VP	MG	M	MP
GIP6	P	G	M	VG	MG	P	VP	MP	G	P	P	MG
GIP7	M	VP	G	G	M	MP	MG	M	MG	VP	MP	P

In addition, the DGI resource of AHEM is shown in Table 4, where “1” indicates that the innovation body has the DGI resource of AHEM and “0” indicates that the innovation body lacks the DGI resource of AHEM. The utilization rate of the DGI resource is presented in “()”. *C*_*11*_ – *C*_*14*_, *C*_*21*_ – *C*_*24*_, and *C*_*31*_ – *C*_*34*_ represent the 12 sub-criteria; *GIP*_1_ – *GIP*_*7*_ represents academic research institutions, and *GBTsR*_*1*_ – *GBTsR*_*8*_ represents the complementary resources.

**TABLE 4:** The status of DGI resources of AHEM.

	GBTsR1	GBTsR2	GBTsR3	GBTsR4	GBTsR5	GBTsR6	GBTsR7	GBTsR8
GBE	1	0	1	1	0	1	0	0
	0.85	0.20	0.85	0.80	0.15	0.70	0.25	0.20
GIP1	0	1	0	0	1	0	1	0
	0.25	0.85	0.25	0.25	0.65	0.20	0.75	0.25
GIP2	0	1	0	1	0	1	1	0
	0.55	0.55	0.25	0.75	0.30	0.65	0.85	0.20
GIP3	1	0	0	1	0	0	1	1
	0.65	0.25	0.30	0.85	0.25	0.50	0.75	0.80
GIP4	0	0	1	1	0	1	0	1
	0.25	0.35	0.75	0.85	0.20	0.80	0.35	0.95
GIP5	0	1	1	0	1	0	1	1
	0.35	0.65	0.75	0.30	0.65	0.35	0.75	0.65
GIP6	0	1	0	1	1	0	0	1
	0.25	0.85	0.35	0.65	0.75	0.20	0.30	0.65
GIP7	0	0	1	0	1	0	1	0
	0.25	0.30	0.65	0.35	0.85	0.25	0.75	0.35

### Results and Discussion

#### Results

(1) The niche-fitness evaluation of academic research institutes

**Step 1:** The two matrices given by DGI and AHEM experts are transformed into two defuzzied matrices based on Table 2, and the two defuzzied matrices are presented in Tables 5, 6.

**TABLE 5:** The matrix of defuzzification given by DGI experts.

	C11	C12	C13	C14	C21	C22	C23	C24	C31	C32	C33	C34
GIP1	0.0055	0.2683	0.2138	0.0690	0.0305	0.2678	0.2915	0.3317	0.1942	0.2162	0.1598	0.2407
GIP2	0.0990	0.0818	0.1210	0.2042	0.2325	0.0910	0.1650	0.2630	0.1388	0.1548	0.0053	0.1362
GIP3	0.2915	0.3167	0.2098	0.1618	0.1318	0.1518	0.3135	0.0442	0.0832	0.0930	0.2232	0.0818
GIP4	0.1650	0.2407	0.0282	0.1152	0.0305	0.2122	0.0385	0.0063	0.1388	0.2728	0.3137	0.2407
GIP5	0.2310	0.1362	0.0728	0.0268	0.2587	0.1518	0.7425	0.0442	0.0047	0.2162	0.4532	0.0818
GIP6	0.0385	0.2407	0.1210	0.2272	0.1842	0.0352	0.0055	0.1130	0.2453	0.0362	0.0373	0.1908
GIP7	0.1650	0.0045	0.2138	0.2042	0.1318	0.0910	0.2310	0.1880	0.1942	0.0052	0.0960	0.0317

**TABLE 6:** The matrix of defuzzification given by AHEM experts.

	C11	C12	C13	C14	C21	C22	C23	C24	C31	C32	C33	C34
GIP1	0.0800	0.2523	0.1650	0.0268	0.1210	0.1618	0.1388	0.1500	0.2812	0.2307	0.0337	0.1702
GIP2	0.0313	0.0337	0.2915	0.1152	0.2380	0.1152	0.0325	0.2100	0.1430	0.0043	0.0048	0.1020
GIP3	0.1330	0.0048	0.2310	0.2042	0.0728	0.0268	0.1942	0.3500	0.1430	0.0780	0.0615	0.0057
GIP4	0.1860	0.1430	0.1650	0.2272	0.1692	0.2042	0.0047	0.0050	0.0048	0.2567	0.2812	0.1702
GIP5	0.2607	0.2523	0.0385	0.0345	0.2138	0.0690	0.2453	0.1500	0.0860	0.2307	0.1430	0.3013
GIP6	0.2345	0.2812	0.0055	0.2042	0.1210	0.2042	0.1388	0.1500	0.2000	0.1302	0.0860	0.2388
GIP7	0.0800	0.0337	0.0990	0.1618	0.0728	0.2272	0.2453	0.2950	0.1430	0.0780	0.2523	0.0057

**Step 2:** A merged matrix is calculated based on the two defuzzied matrices. The rule in the calculation process is that the weight of *C*_*11*_-in the matrix is respectively (0.5, 0.5); the weight of *C*_*21*_ – *C*_*24*_ is respectively (0.6, 0.4); the weight of *C*_*31*_-*C*_*31*_ is respectively (0.4, 0.6). The merged matrix is presented in Table 7. The prospect value matrix of the merged matrix and the weight matrix is calculated, and the results are presented in Tables 8, 9.

**TABLE 7:** The merged matrix based on the matrices of defuzzification.

	C11	C12	C13	C14	C21	C22	C23	C24	C31	C32	C33	C34
GIP1	0.0428	0.2603	0.1894	0.0479	0.0848	0.2042	0.1999	0.2227	0.2290	0.2220	0.1094	0.2125
GIP2	0.0652	0.0578	0.2063	0.1597	0.2358	0.1055	0.0855	0.2312	0.1405	0.0946	0.0051	0.1225
GIP3	0.2123	0.1608	0.2204	0.1830	0.0964	0.0768	0.2419	0.2277	0.1071	0.0870	0.1585	0.0514
GIP4	0.1755	0.1918	0.0966	0.1712	0.1137	0.2074	0.0182	0.0055	0.0852	0.2664	0.3007	0.2125
GIP5	0.2458	0.1943	0.0557	0.0307	0.2318	0.1021	0.4442	0.1077	0.0372	0.2220	0.3291	0.1696
GIP6	0.1365	0.2609	0.0633	0.2157	0.1463	0.1366	0.0855	0.1352	0.2272	0.0738	0.0568	0.2100
GIP7	0.1225	0.0191	0.1564	0.1830	0.0964	0.1727	0.2396	0.2522	0.1737	0.0343	0.1585	0.0213

**TABLE 8:** The prospect value matrix of the merged matrix.

	C11	C12	C13	C14	C21	C22	C23	C24	C31	C32	C33	C34
GIP1	–0.2544	0.0795	0.0418	–0.3237	–0.0903	0.0786	0.0000	0.0000	0.0996	0.1372	–0.1440	0.0526
GIP2	–0.2001	–0.3486	0.0601	–0.0401	0.1322	–0.0963	–0.3032	0.0127	0.0000	0.0000	–0.3923	–0.1389
GIP3	0.0869	–0.0963	0.0749	0.0170	–0.0574	–0.1711	0.0517	0.0079	–0.1026	–0.0280	0.0000	–0.3122
GIP4	0.0484	0.0000	–0.1714	0.0000	0.0000	0.0818	–0.4555	–0.5328	–0.1598	0.1785	0.1512	0.0526
GIP5	0.1200	0.0042	–0.2711	–0.3633	0.1284	–0.1054	0.2434	–0.3046	–0.2771	0.1372	0.1775	0.0000
GIP6	0.0000	0.0801	–0.2530	0.0544	0.0413	0.0000	–0.3032	–0.2394	0.0978	–0.0678	–0.2733	0.0500
GIP7	–0.0477	–0.4357	0.0000	0.0170	–0.0574	0.0453	0.0492	0.0379	0.0420	–0.1726	0.0001	–0.3811

**TABLE 9:** The weight matrix of the merged matrix.

Guidelines	*w*_*j*_	Guidelines	*w*_*j*_	Guidelines	*w*_*j*_
*C*_11_	0.2673	*C*_11_	0.1746	*C*_11_	0.2392
*C*_12_	0.2939	*C*_12_	0.1631	*C*_12_	0.3251
*C*_13_	0.2180	*C*_13_	0.3780	*C*_13_	0.2258
*C*_14_	0.2208	*C*_14_	0.2843	*C*_14_	0.2099
Total	1	Total	1	Total	1

**Step 3:** The computing procedures on three main criteria are conducted based on the VIKOR approach, and the result is presented in Table 10. The weights of the three main criteria are 0.2709, 0.4440, and 0.2851 respectively.

**TABLE 10:** The computing result of academic research institutes.

	*SC*_1_	*RC*_1_	*QC*_1_	*SC*_2_	*RC*_2_	*QC*_2_	*SC*_3_	*RC*_3_	*QC*_3_
*GIP*_1_	0.4883	0.2673	0.8747	0.3272	0.1746	0.1073	0.1657	0.1274	0.0000
*GIP*_2_	0.5321	0.2443	0.8717	0.4230	0.2956	0.4692	0.5470	0.2258	0.6494
*GIP*_3_	0.1440	0.1005	0.0000	0.4305	0.1631	0.1879	0.5664	0.1911	0.5820
*GIP*_4_	0.2806	0.1552	0.3173	0.7660	0.3780	1.0000	0.1751	0.1647	0.1042
*GIP*_5_	0.4821	0.2208	0.7465	0.2943	0.1706	0.0649	0.3029	0.2392	0.4269
*GIP*_6_	0.2923	0.2066	0.4653	0.5578	0.2956	0.6072	0.4091	0.2280	0.5101
*GIP*_7_	0.4806	0.2939	0.9337	0.2774	0.1488	0.0000	0.6418	0.3251	1.0000

(2) The partner selection based on niche field model

**Step 1:** The niche-fitness of academic research institutes shown in Table 11 is obtained by the combined attribute weight method.

**TABLE 11:** The niche-fitness of academic research institutes.

	*GIP*_1_	*GIP*_2_	*GIP*_3_	*GIP*_4_	*GIP*_5_	*GIP*_6_	*GIP*_7_
SC	0.6439	0.2244	0.6029	0.4257	0.5405	0.3301	0.4440
RC	0.3505	0.1613	0.2709	0.2479	0.3886	0.1317	0.4440
QC	0.8502	0.0474	0.6740	0.4260	0.7880	0.1260	0.7618
Ranking	1	7	4	5	2	6	3

**Step 2:** The utilization rate of resources is normalized, and the niche-fitness mass of AHEM enterprises and academic research institutes is calculated. The field strength, attraction, radius, and resistance based on niche-fitness are calculated where *K* = 0.8 and δ = 0.5. Resistance is directly proportional to the amount of resources owned by oneself, which is set to account for 5% of the complementary resources. The above calculation results are shown in Table 12.

**TABLE 12:** The niche mass, field strength, attraction, radius, and resistance.

	*M*		*GIP*_1_	*GIP*_2_	*GIP*_3_	*GIP*_4_	*GIP*_5_	*GIP*_6_	*GIP*_7_
*M*	0.8000	*m*	0.6522	0.6829	0.7011	0.7444	0.7753	0.7250	0.6000
*E*	∞	*E*	0.1973	0.0717	0.1595	0.1202	0.2111	0.0826	0.1565
**F*_*a*_*	∞	**F*_*a*_*	0.1579	0.0573	0.1276	0.0962	0.1689	0.0661	0.1252
*R*	1.0000	*R*	1.1498	1.9526	1.3260	1.5740	1.2120	1.8740	1.2382
*F*_*r*_	0.0000	*F*_*r*_	0.0326	0.0341	0.0351	0.0372	0.0388	0.0363	0.0300
*F*	∞	*F*	0.1252	0.0232	0.0926	0.0589	0.1301	0.0298	0.0952
Ranking			2	7	4	5	1	6	3

**Step 3:** The radius threshold value ε_*T*_ is set to 1.5 based on advice from DGI experts and AHEM experts. The attraction threshold value is set to 0.1409, and the result is based on the experiment (Maximum attraction value *F*_*amax*_ = 0.2280, and then the threshold value ς_*T*_ = 0.2280×0.618 = 0.1409).

**Step 4:** The remaining partners are sorted based on the resultant force, and one or more partners are selected to enter the AHEM system. The standard is that the radius value of the candidate partner’s location must be completely lesser or greater than the threshold value, namely *R*_*T*_≤ε_*T*_, *F*_*r*_≥ς_*T*_ and *F*_*r*_≥*F*_*a*_.

**(i)** The first-round selection based on the radius threshold value.

**FIGURE 4:**
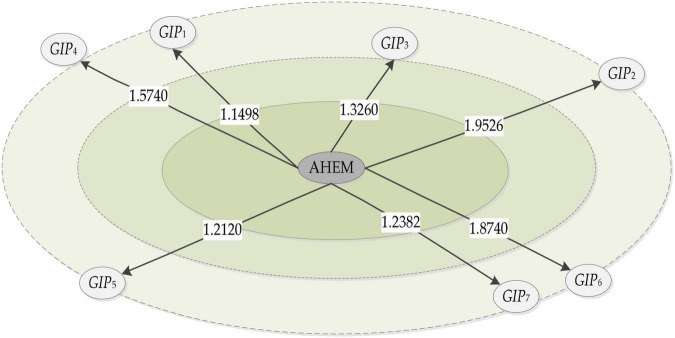
The hierarchical structure of AHEM enterprises and academic institutions.

The hierarchy of AHEM enterprises and academic research institutes is shown in Figure 4.

As can be seen in Figure 4, the radii of *GIP*_1_, *GIP*_*3*_, *GIP*_*5*_ and *GIP*_*7*_ among the hierarchy of medium niche-fitness (1,1.5], and are all less than the radius threshold valueε_*T*_ = 1.5. Hence, *GIP*_2_, *GIP*_*4*_, and *GIP*_*6*_ are eliminated.

**(ii)** The attractive force of *GIP*_1_, *GIP*_*3*_, *GIP*_*5*_ and *GIP*_*7*_ is respectively 0.1579, 0.1276, 0.1689, and 0.1252. The partner *GIP*_*3*_and *GIP*_*7*_ are eliminated based on the attraction threshold value of 0.1409.

**FIGURE 5:**
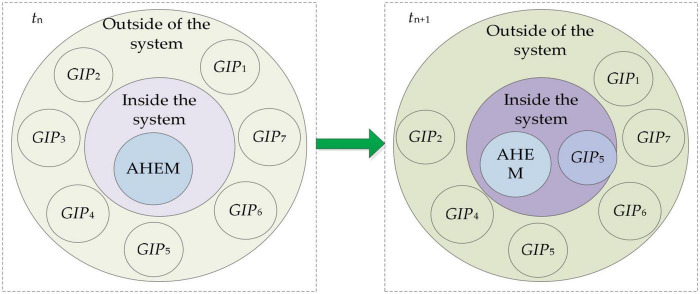
The dynamic selection process.

**(iii)** The attraction of *GIP*_*1*_ and *GIP*_*5*_ is greater than the resistance. *GIP*_*5*_ is the best academic research institute partner, and the dynamic selection process is presented in Figure 5.

This study proposes a theoretical framework based on niche strength and degree of niche overlap for niche-fitness evaluation, and superposition of technology (innovation ability of digital green technology of AHEM, maturity and reliability of digital green technology of AHEM, timeliness of digital green technology of AHEM, compatibility of digital green technology of AHEM), mutual trust (compatibility of organizational atmosphere and values between the two parties, willingness of academic research parties to participate in DGI, integration degree of DGI R&D teams of both sides, and reasonable price of DGI projects), and technical complementarity (complementarity of green innovation technologies for AHEM, complementarity of digital innovation technologies for AHEM, complementarity of the convergence of digital and green technologies, complementary of multi-technology convergence innovation and manufacturing) are reasonably incorporated into the theoretical framework. These factors have important reference value for AHEM enterprises to choose DGI academic research partners. Moreover, this study also theoretically expends the applications of field theory considering resource complementarity with fuzzy linguistic information in the collaborative innovation paradigm.

Based on the above analysis, this study not only proposed a criteria framework based on niche theory for niche-fitness evaluation but also developed a novel niche field model for selecting the DGI partner based on field theory. First, the TFN set theory is used to obtain the cooperation aspiration of academic research institutes according to the criteria framework. Moreover, fuzzy prospect theory is introduced to help AHEM enterprises avoid risks blindly or like risks so that decision-making is more reasonable. Second, the combined method of the VIKOR method and prospect theory is coupled with the combinations of assigned attribute weights calculated to evaluate the niche-fitness of academic research institutes. Furthermore, a novel niche field model considering resource complementarity is developed to select the best academic research institute. Finally, the results of a case study show that the criteria framework and the niche field model can be applied to AHEM enterprises partner selection.

#### Discussion

##### Managerial Implications

This study has two main managerial implications, which can provide some management inspiration for AHEM enterprises who choose DGI partner in the development of agricultural high-end equipment. This study not only proposed a criteria framework based on niche theory for niche-fitness evaluation but also developed a novel niche field model for selecting the management of DGI partner based on field theory. The criteria framework and the novel niche field model can be used to assist AHEM enterprises to perform DGI practice in the development of agricultural high-end equipment. The practical managerial implications of this study are discussed below.

This study proposed a criteria framework based on niche theory for niche-fitness evaluation. DGI has been viewed as one of the key factors affecting the competitive advantages and strategic selection of AHEM enterprises. From the perspective of niche theory, this study proposes a criteria framework with 12 sub-criteria for DGI partner selection of AHEM enterprises. DGI ability superposition of technology, mutual trust, and technical complementarity are beneficial for transferring DGI knowledge from academic research institutes to the AHEM industry. The criteria framework based on niche theory can be applied to real-world partner selection for AHEM DGI in the development of agricultural high-end equipment.

This study also developed a novel and robust framework for DGI partner selection of AHEM enterprises. Managers of AHEM enterprises often have to face the problem of DGI partner selection. This study presents a novel niche field model to help AHEM enterprises select their DGI partners from the masses of academic research institutes. Decision rules and resource complementarity are fully incorporated into the novel niche field model. First, TFN theory is used to obtain the cooperation aspiration of academic research institutes according to the criteria framework. Moreover, fuzzy prospect theory is introduced to help AHEM enterprises avoid risks blindly or like risks, and make decisions more rational. Second, the combined method of the VIKOR method and prospect theory is coupled with the criteria weight calculated by a combination that assigns attribute weights to evaluate the niche-fitness of academic research institutes. Furthermore, a novel niche field model considering resource complementarity is developed to select the best academic research institute. This model can help AHEM enterprises carry out DGI in the development of agricultural high-end equipment.

##### Theoretical Implications

This study proposes a theoretical framework based on niche strength and degree of niche overlap for niche-fitness evaluation, and the superposition of technology, mutual trust, and technical complementarity are reasonably incorporated into the theoretical framework. Moreover, this study also theoretically expends the applications of field theory considering resource complementarity with fuzzy linguistic information in the collaborative innovation paradigm.

In this study, field theory is introduced to reflect the resource complementarity, rationality, and matching of the partner selection process for DGI, and niche theory is introduced to reflect the potential of academic research institutes for collaborative innovation. This study provided a novel theoretical framework that can help AHEM enterprises select their DGI innovation partners from the masses of academic research institutes in the development of DGI. The combined method of TFN theory and VIKOR method with objective weight is coupled with the prospect theory. The criterion weight is calculated by combining the weight of combination attributes to evaluate the niche-fitness of academic research institutes. And then a niche field model considering resource complementarity is developed to select the best academic research institute. Finally, the analytical results of a case study show that the criteria framework and the novel niche field model can be applied to real-world partner selection for AHEM enterprises and yield selecting results that are more realistic.

## Conclusion and Implications

### Conclusion

Although the agricultural economy drives DGI for the development of agricultural high-end equipment, partner selection for DGI is still plagued with various barriers. The R&D and application of facility agricultural equipment technology are still not as mature as developed countries, and the agricultural equipment industry is quite different from foreign countries. It requires AHEM enterprises to actively strengthen the DGI of AHEM enterprises on the basis of which to improve the technical level of China’s facility agricultural equipment. Cooperation between AHEM enterprises and academic research partners can accelerate the R&D efforts of AHEM enterprises and promote the integrated innovation of agricultural technological achievements. This helps improve the ability of the successful transformation of agricultural science and technology. Therefore, it is of great significance to strengthen the research on cooperative DGI partner selection for promoting enterprises to implement cooperative DGI mode and improve the DGI innovation ability of AHEM enterprises. In the present study, a niche fitness evaluation standard framework based on niche theory was proposed and a niche field model for innovation partner selection management based on niche theory was established. The standard framework and novel niche field model can help enterprises carry out DGI in the development of high-end agricultural equipment.

The results are as follows: (i) This study not only proposed a criteria framework based on niche theory for niche-fitness evaluation but also developed a novel niche field model for selecting the management of DGI partner based on field theory. The theoretical framework based on niche strength and degree of niche overlap includes superposition of technology, mutual trust, and technical complementarity. (ii) In the niche field model, fuzzy prospect theory is introduced to help AHEM enterprises avoid risks blindly or like risks. The combined method of the VIKOR method with objective weight is coupled with the prospect theory. The criterion weight is calculated by combining the weight of combination attribute to evaluate the niche-fitness of academic research institutes. (iii) The niche field model has been successfully applied to practical cases to illustrate how the model can be implemented to solve the problem of DGI partner selection. The results of a case study show that the criteria framework and the niche field model can be applied to real-world partner selection for AHEM enterprises.

### Implications

The study has the following theoretical and practical implications: (i) constructing a criteria framework based on niche theory; (ii) developing a novel niche field model for DGI partner selection of AHEM enterprises; and (iii) assisting AHEM enterprises to perform DGI practice. In addition, this study theoretically expends the applications of field theory considering resource complementarity with fuzzy information in the DGI paradigm. The criteria framework and the novel niche field model can be used to assist AHEM enterprises to perform DGI practice in the development of agricultural high-end equipment. AHEM enterprises should strengthen the DGI ability and investment to promote the combination of industry-university-research and improve the conversion rate of digital green achievements. The government should promote the effective integration of resources, such as intelligence, interdisciplinary knowledge, large-scale scientific research facilities, and information so that scientists and technology teams in large projects can fully share resources. The cooperative innovation platform of universities and scientific research institutes with AHEM enterprises as the main body should be established to provide solid material and equipment guarantee for agricultural mechanization to enter the advanced stage and agricultural modernization.

### Deficiencies and Future Prospects

In this study, there are still some limitations that deserve the attention of future research. AHEM enterprises can be classified according to R&D scale or enterprise scale, and the classified AHEM enterprises are used to carry out case studies. In addition, artificial intelligence technologies (AIs) are gradually applied to decision-making problems, and the combination of resource complementarity and AIs play an important role in enlightenment in the future.

## Data Availability Statement

The raw data supporting the conclusions of this article will be made available by the authors, without undue reservation.

## Author Contributions

SY and JX: conceptualization. SY: methodology, software, and writing—review and editing. YW and SY: validation. YW: writing—original draft preparation. All author: read and agreed to the published version of the manuscript.

## Conflict of Interest

The authors declare that the research was conducted in the absence of any commercial or financial relationships that could be construed as a potential conflict of interest.

## Publisher’s Note

All claims expressed in this article are solely those of the authors and do not necessarily represent those of their affiliated organizations, or those of the publisher, the editors and the reviewers. Any product that may be evaluated in this article, or claim that may be made by its manufacturer, is not guaranteed or endorsed by the publisher.
